# Advances and challenges in nutritional screening and assessment for cancer patients: a comprehensive systematic review and future directions

**DOI:** 10.3389/fnut.2025.1688344

**Published:** 2025-10-16

**Authors:** Luocheng Zhang, Zibo Ding, Yanfei Zhao, Ziyao Cheng, Jiahao Hu, Lanqing Huo

**Affiliations:** ^1^West China Medical School, West China Hospital, Sichuan University, Chengdu, China; ^2^College of Life Sciences, Sichuan University, Chengdu, China; ^3^College of Biomedical Engineering, Sichuan University, Chengdu, China; ^4^State Key Laboratory of Oncology in South China, Department of Radiation Oncology, Guangdong Provincial Clinical Research Center for Cancer, Sun Yat-sen University Cancer Center, Guangzhou, China

**Keywords:** cancer, malnutrition, malnutrition assessment, malnutrition screening tools, targeted therapy

## Abstract

**Introduction:**

Cancer-associated malnutrition is a pervasive and under-recognized complication that profoundly impacts treatment tolerance, clinical outcomes, and quality of life. Despite the availability of multiple nutritional screening and assessment tools, these instruments differ widely in sensitivity, specificity, and ease of integration into clinical workflows, and no universally accepted standard exists. This review critically examines the current landscape of malnutrition assessment in oncology, summarizes tool performance across populations and cancer types, and proposes strategies—such as artificial intelligence–enabled models and internationally harmonized protocols—to improve diagnosis, treatment planning, and overall patient outcomes.

**Methods:**

A comprehensive literature search was conducted across PubMed, Web of Science, Embase, and Elsevier databases, covering studies published up to 13 March 2025. Medical Subject Headings (MeSH) were used to identify terms including “malnutrition,” “cachexia,” “cancer,” “nutritional status assessment,” “nutritional screening,” and “nutritional screening tool.” Boolean operators refined the strategy, and a two-stage screening excluded studies with irrelevant populations, outcomes, or designs, as well as non-peer-reviewed sources.

**Results:**

Significant heterogeneity was found in tool performance and applicability across cancer types, clinical settings, and demographic subgroups. General instruments such as the Malnutrition Universal Screening Tool (MUST) and Nutritional Risk Screening 2002 (NRS-2002) demonstrated strong predictive validity in broad clinical use, whereas condition-specific tools like Patient-Generated Subjective Global Assessment (PG-SGA) offered superior sensitivity in high-risk populations, including patients with gastric or head and neck cancers. However, variability in thresholds, assessment frequency, and validation approaches highlights the urgent need for standardization.

**Discussion:**

Current assessment strategies are limited by subjectivity, static single-point evaluations, and inconsistent implementation. Future innovations should integrate artificial intelligence, dynamic longitudinal monitoring, and multimodal data analytics to develop objective and personalized evaluation systems. Establishing globally harmonized standards will be crucial to improving nutritional care, reducing malnutrition-related morbidity, and enhancing survival and quality of life for patients with cancer.

## Highlights

• Rigorous head-to-head evaluation of nutritional tools: this review delivers a comprehensive, side-by-side assessment of widely used nutritional screening instruments, clarifying their diagnostic accuracy and clinical utility for identifying cancer-related malnutrition.
• Precision guidance for cancer-specific care: we provide actionable recommendations for selecting the most appropriate tools tailored to cancer type and patient demographics, supporting precision nutrition strategies in oncology.
• Exposing critical gaps in current practice: our analysis underscores major shortcomings—including subjectivity, static single-point assessments, and lack of standardized protocols—that limit real-world implementation.
• A roadmap for next-generation solutions: we call for AI-enabled predictive models, dynamic longitudinal monitoring, and internationally harmonized standards to transform nutritional assessment and optimize cancer outcomes.

## Introduction

Malnutrition is a prevalent and clinically significant comorbidity among patients with cancer, affecting a substantial proportion of individuals across disease types and care settings (1, 2). Comprehensive assessments indicate that moderate to severe malnutrition is common, with prevalence rates reaching approximately 25% in patients with gastroenteropancreatic neuroendocrine tumors (GEP-NETs) (3). Similar trends are observed in gynecologic malignancies, particularly in advanced or recurrent disease, where malnutrition and sarcopenia are frequently documented (4).

**FIGURE 1:**
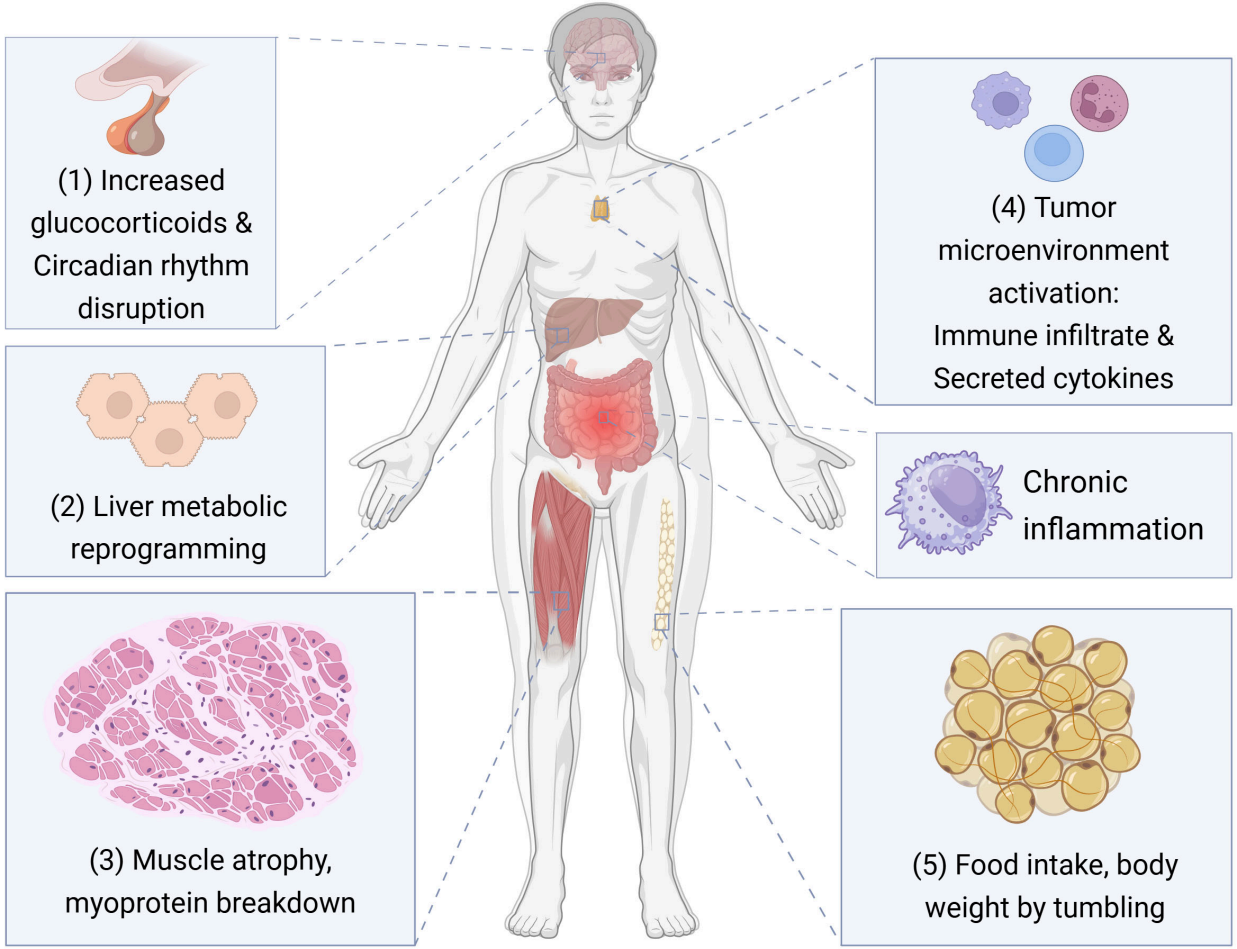
The impact of cancer cachexia at the tissue and organ level. This figure illustrates the key pathophysiological mechanisms through which cancer cachexia affects various tissues and organ systems. The processes include: (1) elevated glucocorticoid levels and disruption of circadian rhythms; (2) metabolic reprogramming in the liver; (3) skeletal muscle atrophy and enhanced myoprotein breakdown; (4) activation of the tumor microenvironment, characterized by immune cell infiltration and pro-inflammatory cytokine secretion; (5) systemic chronic inflammation. These factors collectively contribute to reduced food intake and progressive loss of body weight, hallmark features of cancer cachexia.

Cancer-associated malnutrition profoundly influences treatment tolerance, recovery trajectories, and overall health status (5, 6). It is typically characterized by involuntary weight loss with significant depletion of skeletal muscle and adipose tissue (7, 8). This progressive tissue wasting disrupts organ function and induces a fragile yet metabolically stable state (9). Closely related to this process is cancer cachexia, a multifactorial syndrome defined by involuntary muscle and fat loss combined with systemic inflammation (7) (Other effects of cancer cachexia are shown in Figure 1). Cancer cachexia is commonly classified into three stages—pre-cachexia, cachexia, and refractory cachexia—reflecting the escalating severity of metabolic and functional impairment (10–12).

The consequences of malnutrition and cachexia extend beyond nutritional metrics, contributing to diminished quality of life, prolonged hospitalization, increased readmission rates, and higher mortality (5, 6). Early identification and systematic assessment of nutritional status have therefore become essential components of comprehensive cancer care, with the goal of improving treatment outcomes and patient wellbeing.

Multiple nutritional screening and assessment tools are currently employed in clinical practice, including the Patient-Generated Subjective Global Assessment (PG-SGA), Mini Nutritional Assessment (MNA), Nutritional Risk Screening 2002 (NRS-2002), and the Malnutrition Universal Screening Tool (MUST). Furthermore, in order to adapt to different clinical environments, nutritional Assessment tools will also extend many variations, such as Mini-Nutritional Assessment Short-Form (MNA-SF), which provides a more convenient, fast and low-cost assessment method (13). However, disparities in sensitivity, specificity, usability, and cultural adaptability remain, and the absence of universally accepted diagnostic criteria has hindered validation efforts, with many studies relying on suboptimal reference standards (e.g., alternative tools, biochemical markers, or composite scores) (14–16).

Given these limitations, a critical synthesis of available screening and assessment methods is urgently needed to guide clinicians in selecting tools tailored to specific cancer types, treatment stages, and patient populations. In addition, the integration of effective nutritional interventions has demonstrated the potential to improve clinical outcomes and quality of life (17, 18). This review provides a comprehensive comparison of existing malnutrition screening and assessment strategies, offering evidence-based guidance for optimizing nutritional management in oncology.

## Methods

We conducted a systematic literature review to evaluate nutritional screening and assessment tools in oncology populations, employing a hybrid methodology that integrates the Preferred Reporting Items for Systematic Reviews and Meta-Analyses (PRISMA) 2020 guidelines with established Systematic Literature Review (SLR) principles. The review process comprised four key stages: (1) formulation of research questions and objectives; (2) definition of review scope; (3) literature search, selection, and eligibility screening; and (4) quality appraisal and validation of included studies.

The primary research question guiding this review was: *Which nutritional screening and assessment tools are commonly used in cancer care, and how do they perform in terms of sensitivity, specificity, and clinical feasibility across diverse healthcare settings?* To address this question, we set the following objectives: (1) systematically identify studies reporting the use of nutritional screening and assessment tools in oncology; (2) classify and compare tools by design, intended application, and target population; (3) evaluate the methodological quality of included studies using appropriate appraisal frameworks; and (4) identify key gaps, challenges, and opportunities to inform future innovation.

A systematic search of PubMed, Web of Science, Embase, and Elsevier databases was performed, incorporating Medical Subject Headings (MeSH) terms from the United States National Library of Medicine (accessed 13 March 2025). Search descriptors included “malnutrition,” “cachexia,” “cancer,” “nutritional status assessment,” “nutritional screening,” and “nutritional screening tool,” combined with Boolean operators (AND and OR) to maximize sensitivity and specificity. No date restrictions were imposed; however, emphasis was placed on studies published within the past 5 years to ensure relevance.

**FIGURE 2:**
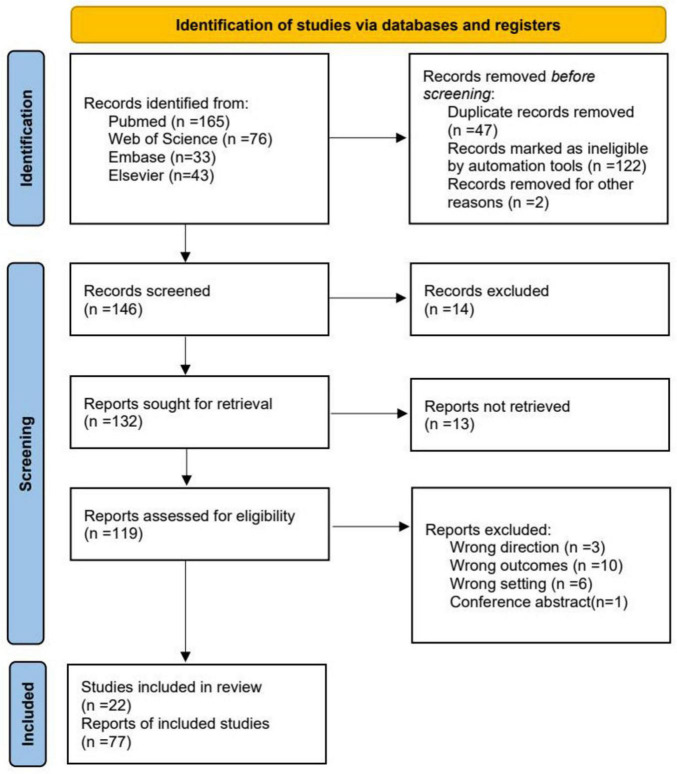
Preferred Reporting Items for Systematic Reviews and Meta-Analyses (PRISMA) flowchart of the study selection process for the systematic review. PRISMA provides authors with guidance and examples of how to completely report why a systematic review was done, what methods were used, and what results were found.

The selection process adhered to PRISMA 2020 standards (Figure 2). A total of 317 records were retrieved. After removing duplicates (*n* = 47) and automated exclusions (*n* = 122), 146 articles were screened. Fourteen were excluded as irrelevant, and full-text retrieval was attempted for 132 articles, of which 13 could not be accessed. Ultimately, 119 articles were assessed for eligibility, and 22 met the inclusion criteria. Exclusion reasons included irrelevant focus (*n* = 3), unsuitable outcomes (*n* = 10), inappropriate settings (*n* = 6), and conference abstracts (*n* = 1). To ensure comprehensiveness, reference lists of relevant reviews were manually screened using Artificial intelligence-assisted literature tools.

This review focused exclusively on nutritional screening and assessment instruments validated or applied in oncology care. Both general-purpose tools (e.g., MUST, NRS-2002, PG-SGA) and cancer-specific instruments were included, spanning inpatient, outpatient, and home-based care settings. Studies involving adult and pediatric oncology patients were considered, with particular attention to tools demonstrating cross-context applicability.

## Results

Nutritional screening and assessment tools demonstrated varied applications and performance in oncology settings. The Malnutrition Universal Screening Tool (MUST) showed high sensitivity (80.0%) and specificity (74.7%), outperforming tools such as NRS-2002 and MNA-SF in certain studies. The Nutritional Risk Screening 2002 (NRS-2002), widely adopted in hospitals, exhibited high sensitivity and reliability in identifying nutritional risk and predicting clinical outcomes such as complications and mortality. The Global Leadership Initiative on Malnutrition (GLIM) criteria offered a sensitive and specific framework for diagnosing disease-related malnutrition using phenotypic and etiologic criteria, applicable across diverse clinical and global settings. Meanwhile, the Patient-Generated Subjective Global Assessment (PG-SGA), tailored specifically for cancer patients, enabled detailed evaluation through patient-reported and clinician-assessed components, facilitating graded interventions based on total score. It demonstrated high clinical utility in identifying malnutrition and guiding nutritional support in oncologic populations.

### Nutrition screening and assessment tools

Nutritional intervention is a critical component of cancer care, with preemptive assessment and management significantly improving surgical outcomes and quality of life (19, 20). Despite its importance, significant gaps exist in clinical implementation. Studies demonstrate that while technology-assisted systems can achieve high screening completion rates [e.g., 91% shortly after admission (21)], only a minority of healthcare professionals (14%) routinely use validated screening tools, despite most (65%) acknowledging the importance of nutrition (22). This gap underscores substantial implementation barriers.

According to ESPEN guidelines, nutritional management involves two key steps: initial screening to identify risk without etiological analysis, followed by a comprehensive assessment for those who screen positive to diagnose malnutrition and develop individualized interventions (23, 24). Table 1 outlines the ESPEN Cancer Nutrition Screening and Assessment Framework. The ESPEN 2003 Guidelines recommend Nutritional Risk Screening 2002 (NRS-2002) as the preferred screening tool for inpatients, where a score ≥ 3 indicates “nutritional risk” and necessitates further assessment (23).

**TABLE 1:** European Society for Clinical Nutrition and Metabolism (ESPEN) cancer nutrition screening and assessment framework (2).

ESPEN cancer nutrition framework			
Component	B1-1: screening	B1-2: nutritional assessment	Key clinical expansions
Core Action	Regular monitoring from diagnosis:	Quantitative evaluation for screen-positive patients:	Mandatory workflow: screening → risk stratification → comprehensive assessment
	• Dietary intake	• Nutrient intake	
	• Weight change	• Nutrition impact symptoms (NIS)	
	• BMI	• Muscle mass	
		• Physical function	
		• Systemic inflammation	
Recommendation	Strong	Strong	Grade A = essential clinical standard
Evidence level	Very low	Very low	Primarily expert consensus (ESPEN Delphi 2023)
Tools	• PG-SGA short form	• Muscle mass: CT (L3-SMI)/BIA	PG-SGA score ≥ 4 triggers full assessment
	• NRS-2002 (inpatients)	• Symptoms: PG-SGA professional part	
		• Inflammation: CRP + IL-6	
		• Function: Handgrip + 4-m walk test	
Critical thresholds	• Weight loss: > 5%/3 months	• Sarcopenia: SMI: male < 52.4 cm^2^/m^2^; female < 38.5 cm^2^/m^2^ (CT)	Single threshold breach = positive screen
	• BMI: < 18.5	• Inflammation: CRP > 10 mg/L	
	• Intake reduction: < 50% of needs	• Function decline: Handgrip M < 28 kg F < 18 kg	
Research gaps	Correlation between screening results and survival outcomes	Predictive validity of assessment tools for treatment tolerance	Priority studies: • AI-driven screening models
			• Muscle dynamics as prognostic biomarker

PG-SGA, Patient-Generated Subjective Global Assessment; NRS-2002, Nutritional Risk Screening 2002; BMI, body mass index, This table outlines the ESPEN cancer nutrition screening and assessment framework. The framework mandates a workflow from screening to risk stratification to full assessment, with specific critical thresholds provided.

### Nutrition screening: quickly and efficiently identify people at risk of malnutrition

The Malnutrition Universal Screening Tool (MUST) is a rapid, repeatable universal screening tool for adult patients in different healthcare settings. The score is based on the following three indicators (24): BMI: 0 (> 20 kg/m^2^), 1 (18.5–20 kg/m^2^), 2 (< 18.5 kg/m^2^); Involuntary weight loss: 0 points (< 5%), 1 point (5%–10%), 2 points (> 10%); Effects of acute illness on food intake: 0 (none), 2 (presence of acute illness that prevents eating for the next 5 days). The total score classified patients as low risk (0), medium risk (1), and high risk (≥ 2) [Cortes et al. (25)]. MUST show the highest sensitivity (80.0%) and specificity (74.7%) in the study, with a positive predictive value of 44.4% (26). Compared to the ESPEN standard as the gold standard, MUST outperforms other tools such as NRS-2002, Malnutrition Screening Tool (MST), Simplified Nutritional Assessment Questionnaire (SNAQ), and MNA-SF on these indicators (27, 28). In addition, MUST is recommended for use in community and hospital settings to be able to predict length of stay and likelihood of readmission and to monitor changes in nutritional status (29, 30).

Kondrup et al. (23) developed the nutrition screening tool NRS-2002 based on 128 studies on the effectiveness of nutritional support (31). At present, it is the most commonly used malnutrition screening tool for hospital patients (32), with high sensitivity, consistent with the diagnosis of experienced physicians, and can also predict adverse outcomes such as complications, mortality, and prolonged hospital stay (33, 34). NRS-2002 is divided into the preliminary screening stage and the scoring stage (35). Initial screening stage: includes 4 questions (BMI < 20.5, weight loss in the past 3 months, recent reduction in intake, serious illness). If either answer is “yes,” formal screening is initiated. This was followed by a scoring stage: divided into nutritional status (0–3 points), disease severity (0–3 points), and age adjustment: patients ≥ 70 years old plus 1 point. Finally, the total score is calculated: nutritional status score + disease score + age score. A total score of ≥ 3 indicates a high risk or definite malnutrition and requires nutritional support (1). The European Society for Clinical Nutrition and Metabolism (ESPEN) recommends that NRS-2002 be used in hospitalized patients, especially those with severe illness, and that NRS-2002 be used in combination with the modified NUTRIC Score (36, 37).

Furthermore, MUST and MST are widely used in various clinical settings. MUST is favored for its ease of use, particularly among emergency and newly diagnosed patients. Lima et al. (38) pointed out that MUST exhibit high sensitivity and accuracy in screening for nutritional risks, making it suitable for the early identification of malnutrition risks among hospitalized patients (38). In studies on cancer patients, MUST and NRS-2002 performed well in predicting clinical outcomes, especially in critical care settings (39). Table 2 provides a comparative overview of various nutritional assessment and screening tools used in clinical practice.

**TABLE 2:** Nutritional assessment tools comparison.

Comparison of nutritional assessment tools					
Tool	Full name	References	Principle	Common formula/components	Features
COUNT	Total lymphocyte count	1970s	Malnutrition leads to reduced lymphocytes	TLC = WBC × lymphocyte%; < 1500/mm^3^ = risk	Quick, non-specific; affected by inflammation (86)
PNI	Prognostic nutritional index	Onodera et al. (87)	Reflects immune-nutritional status	PNI = 10 × albumin (g/dL) + 0.005 × lymphocyte count (/mm^3^)	Cancer, surgery, chronic illness (88)
PINI	Prognostic inflammatory and nutritional index	Pressac et al. (89)	Combines inflammation and nutrition for prognosis	PINI = (α1-acid glycoprotein × CRP)/(prealbumin × transferrin)	Chronic disease, renal failure, cancer (90)
NRI	Nutritional risk index	Magnano et al. (91)	Estimates the surgical risk due to malnutrition	NRI = 1.519 × albumin (g/L) + 41.7 × (current weight/ideal weight)	Used in surgical settings, especially GI (92)
MST	Malnutrition screening tool	Ferguson et al. (93)	The combination of nutrition screening questions with the highest sensitivity and specificity at predicting SGA	Including weight loss, reduced appetite, etc.,	It is simple, efficient and sensitive, strongly predicting the nutritional status defined by SGA (93)
NRS-2002	Nutritional risk screening 2002	Kondrup et al. (23)	To identify under-nourished patients who would probably respond adequately to nutritional support.	First, it evaluates impaired nutritional status on a scale of 0–3 points based on: Weight loss over the past 3 months, Low body mass index (BMI) adjusted for age and reduced dietary intake. Second, it assigns 0–3 points for disease severity by classifying conditions as: Mild (e.g., chronic diseases with minor symptoms: 1 point), Moderate (e.g., major abdominal surgery, stroke, severe pneumonia: 2 points) and Severe (e.g., ICU patients on mechanical ventilation, advanced cancer: 3 points). Third, an age adjustment adds 1 point for patients aged 70 years or older (94).	Greater sensitivity and specificity are reported versus other screening tools in critically ill patients (64, 95)
MUST	Malnutrition universal screening tool	Elia and British Association for Parenteral and Enteral Nutrition (BAPE) (26)	A Popular screening tool in UK national surveys of malnutrition	Based on an older person’s BMI, history of unintentional weight loss, and the probability of future weight loss based on acute disease	Quick, easy (96)
SNAQ	Simplified nutritional appetite questionnaire	Wilson et al. (97)	Predicts risk of weight loss over 6 months	4 questions: appetite, satiety, food taste, number of meals; score < 14 = at risk	Quick, simple; elderly/hospital patients (98)
GNRI	Geriatric nutritional risk index	Bouillanne et al. (99)	Modified NRI for elderly patients	GNRI = 1.489 × albumin (g/L) + 41.7 × (current/ideal weight)	Simple, accurate; elderly inpatient mortality risk (88)
NUTRIC	Nutrition risk in the critically ill	Alberda et al. (100) (mod. 2011)	Assesses nutritional risk and potential benefit from intervention in ICU patients	Age + APACHE II + SOFA + Comorbidities + Pre-ICU nutrition status (IL-6 excluded in modified version)	ICU-specific; Score ≥ 5 = high nutritional risk (101, 102)
MIRT	Malnutrition inflammation risk tool	Jansen et al. (103)	Inflammatory bowel diseases: assessing malnutrition-inflammation synergy in chronic diseases	BMI, weight Loss, CRP	Validated for research, limited routine clinical use (103)
BULT	BMI–lymphocyte–uric acid–triglyceride	Xu et al. (104)	Esophageal squamous cell carcinoma	BMI, lymphocyte, uric acid, and triglyceride	Validation studies are ongoing (104)
DMS	Dialysis malnutrition score	Hassanin et al. (105)	Dialysis patients quantify malnutrition severity in chronic illness	Similar to PS-SGA	Requires clinician judgment; lacks standardization (105)

A quasi-systematic review was performed; therefore, results do not represent an exhaustive search of the literature. BMI, body mass index. This table provides a comparative overview of various nutritional assessment and screening tools used in clinical practice. It includes each tool’s full name, originator, year of development, underlying principle, key components or calculation formula, and distinctive features.

### Nutritional assessment: comprehensive diagnosis of nutritional status and development of personalized intervention programs

Global Leadership Initiative on Malnutrition (GLIM) is a relatively new, widely adaptable tool with higher sensitivity and specificity to disease-related malnutrition (40). Table 3 outlines the standardized two-step process for diagnosing malnutrition in GLIM. The diagnosis of GLIM is based on five criteria, including three phenotypic criteria and two pathological criteria. The three phenotypic criteria are involuntary weight loss (weight loss > 5% within six months or weight loss > 10% for more than six months), low BMI (adult < 18.5 kg/m^2^), muscle mass reduction (grip strength measurement, male < 28 kg, female < 18 kg); the two pathological criteria are reducing food intake or undernutrition (food intake < 50% requirement, lasting > 1 week), inflammation or disease burden (burns, severe infections and other systemic inflammatory reactions) (34). At the time of diagnosis, the patient should meet at least one phenotype standard and one pathological standard at the same time. At the same time, the phenotypical criteria of GLIM can also be used to assess the severity of malnutrition, which is divided into moderate (stage 1) and severe (stage 2) (41). In addition, the scope of application of GLIM is particularly wide and applicable to global clinical environments, including inpatients, community medical care, and chronic disease management (41).

**TABLE 3:** GLIM malnutrition diagnostic framework.*

Criteria category	Diagnostic components	Thresholds and definitions	Assessment methods
Phenotypic criteria	1. Unintentional weight loss	- > 5% within past 6 months	Medical records/patient recall; calibrated scales
		- > 10% beyond 6 months	
	2. Low BMI	- < 18.5 kg/m^2^ (<70 years)	Height/weight measurement (adjust for edema)
		- < 20.0 kg/m^2^ (≥70 years)	
		- < 17.0 kg/m^2^ (severe)	
	3. Reduced muscle mass	- ASMI: M <7.0 kg/m^2^; F <5.7 kg/m^2^	Gold standard: DEXA/CT/MRI
		- Grip strength: M <28 kg; F <18 kg	Bedside: BIA/hand dynamometer
		- Calf circumference: <31 cm	Field: anthropometry
Etiologic criteria	1. Reduced intake/absorption	- Energy intake <50% of needs for >1 week	24-h dietary recall; clinical evaluation of GI function
		- Malabsorption (e.g., IBD, SBS, chronic diarrhea)	
	2. Inflammation/disease burden	- Acute: sepsis, trauma, major surgery	Lab tests
		- Chronic: CRP ≥10 mg/L, active cancer, autoimmune flares	Disease activity indices

*Required: Positive nutrition screening (e.g., NRS-2002 ≥3, MUST ≥1, MNA-SF ≤11) before assessment. This table outlines the standardized two-step process for diagnosing malnutrition. Specific diagnostic thresholds and recommended assessment methods for each component are provided. The Global Leadership Initiative on Malnutrition (GLIM) criteria provide a consensus-based, global framework for the diagnosis and severity grading of malnutrition in clinical populations.

Patients-Generated Subjective Global Assessment (PG-SGA) is a further development of Subjective Global Assessment (SGA), a nutritional assessment tool specially tailored for tumor patients (42). Table 4 outlines the components and criteria of the Subjective Global Assessment (SGA). The evaluation of PG-SGA is based on two parts: patient self-assessment and professional evaluation. The patient’s self-assessment includes weight changes, dietary intake, and symptoms affecting eating and mobility, with a score range of 0–13 points; professional evaluation includes metabolic needs, disease-related stress, and physical examination with a total score of 0–7 (43, 44). The standard of testing is that when the total score is greater than or equal to 9 points, it is severe malnutrition. A total of 2–8 points prompt the need to adjust the diet or oral nutrition. Regular testing is enough when 0–1 point is enough. At the same time, it should be noted that some guidelines suggest that when the PG-SGA score in tumor patients is > 4, it indicates nutritional risks (45). It is characterized by hierarchical intervention based on the total score to assess health status, demonstrating high sensitivity and accuracy. Its effectiveness significantly exceeds that of GLIM in the treatment of certain tumors (46).

**TABLE 4:** SGA (subjective global assessment) framework.

Subjective global assessment		
Component	Assessment criteria	Scoring/grading
**1. Medical history**		
- Weight change	- % loss in 6 months; pattern (steady/sudden)	> 10% loss = significant
- Dietary intake	- Change vs. usual intake (duration/severity)	< 50% intake > 2 weeks = significant
	Type: liquid/full diet, supplements	
- GI symptoms	- Nausea/vomiting, diarrhea, anorexia (> 2 weeks)	Persistent symptoms = significant
- Functional capacity	- Energy levels, ambulation status (bedrest/ambulatory)	Bedridden/severely limited = significant
- Disease and metabolic	- Diagnosis (e.g., cancer, IBD)	High metabolic demand = significant
	- Stress factors (fever, wounds)	
**2. Physical examination**		
- Subcutaneous fat	Loss over triceps/chest (mild/moderate/severe)	Moderate-severe loss = abnormal
- Muscle wasting	Temples, clavicles, scapulae, quadriceps, interossei	Obvious wasting = abnormal
- Edema	Ankle/sacral edema	Present = abnormal
- Ascites	Abdominal fluid	Present = abnormal
**3. Global rating**		
- SGA category	Combines history + physical findings	A: Well-nourished
		B: Moderately malnourished
		C: Severely malnourished

This table outlines the components and criteria of the subjective global assessment (SGA), a clinician-rated tool that integrates medical history and physical examination to assess nutritional status.

In summary, various nutritional screening tools have their advantages in clinical applications. Choosing the appropriate tool should consider the specific conditions of patients and the clinical environment. Future research should further explore the adaptability and effectiveness of these tools in different types of cancer patients to optimize nutritional management strategies for cancer patients.

## Discussion

Current screening and assessment tools for cancer-related malnutrition show considerable variability in effectiveness and consistency across clinical contexts. Ruan et al. (47) employed hierarchical Bayesian latent class meta-analysis to compare three tools—MNA, NRS-2002, and PG-SGA—and demonstrated that PG-SGA had superior diagnostic accuracy, achieving sensitivity and specificity of 96.4% and 90.5%, respectively (2). In contrast, MNA achieved a sensitivity of 91.0% and specificity of 72.0%, while NRS-2002 exhibited lower sensitivity (74.7%) but higher specificity (85.4%) (47). Table 5 details the full 18-item MNA. These findings highlight PG-SGA’s value for early detection of malnutrition risk, particularly in patients requiring urgent intervention. Similarly, Gascón-Ruiz et al. (48) reported that the GLIM criteria significantly outperformed ESPEN standards in detecting malnutrition (46.7% vs. 21.2%) (48), reinforcing the importance of evidence-based tool selection tailored to patient conditions (49).

**TABLE 5:** MNA full assessment (18 items).

Mini Nutritional Assessment			
Domain	Item	Scoring criteria	Points
A. Anthropometry	1. BMI (kg/m^2^)	> 23.0: 3 ● 21.0–23.0: 2 ● < 21.0: 1	0–3
	2. Calf circumference (cm)	≥ 31: 3 ● < 31: 0 (exclude edema)	0–3
	3. Weight loss (past 3 months)	None: 3 ● 1–3 kg: 2 ● > 3 kg: 0	0–3
B. Dietary intake	4. Meals per day	3 meals: 2 ● 2 meals: 1 ● 1 meal: 0	0–2
	5. Protein intake (dairy/meat/legumes)	≥ 2 servings/day: 2 ● 1 serving: 1 ● none: 0	0–2
	6. Fruit/vegetable intake	≥ 2 servings/day: 2 ● 1 serving: 1 ● none: 0	0–2
	7. Fluid intake (cups/day)	> 5: 2 ● 3–5: 1 ● < 3: 0	0–2
	8. Feeding autonomy	Independent: 2 ● needs help: 1 ● dependent: 0	0–2
C. Health status	9. Acute illness/stress (past 3 months)	None: 3 ● Mild (e.g., cold): 2 ● moderate (e.g., pneumonia): 1 ● severe (e.g., stroke): 0	0–3
	10. Neuropsychological issues	None: 3 ● Mild dementia/depression: 2 ● Moderate: 1 ● Severe: 0	0–3
	11. Pressure ulcers/skin wounds	None: 2 ● present: 0	0–2
D. Function	12. Mobility	Independent: 3 ● uses aid: 2 ● wheelchair/bedbound: 0	0–3
	13. Cognition (MMSE ≥ 24)	Normal: 2 ● mild impairment: 1 ● moderate/severe: 0	0–2
	14. ADLs (dressing/washing)	Independent: 2 ● partial help: 1 ● dependent: 0	0–2
E. Self-assessment	15. Self-view of nutritional status	Good: 3 ● Fair: 2 ● poor: 0	0–3
	16. Self-view of health	Good: 2 ● Fair: 1 ● poor: 0	0–2
	17. Mid-arm circumference (MAC, cm)	> 26: 1 ● ≤ 26: 0.5	0–1
	18. Calf circumference (CC, cm)	> 31: 1 ● ≤ 31: 0	0–1
Total score			0–30
**Interpretation and action**			
Total score	Nutritional status	Clinical action	
24–30	Normal	Preventive education	
17–23.5	At risk of malnutrition	Dietary counseling+ oral nutritional supplements (ONS)	
< 17	Malnourished	Aggressive intervention (enteral/parenteral nutrition) + treat underlying causes	

This table presents the complete 18-item Mini Nutritional Assessment (MNA) tool, validated for older adults (≥ 65 years) and endorsed by ESPEN. The assessment covers five domains: (A) Anthropometry, (B) Dietary Intake, (C) Health Status, (D) Function, and *E) Self-Assessment.

### Cancer-specific applications

Tool performance varies by cancer type. In colorectal cancer, MUST demonstrated significant associations with body composition metrics and clinical outcomes (50). Its use of BMI (< 18.5 kg/m^2^) and weight loss (> 5%) aligns closely with common nutritional deterioration patterns. Xie et al. (51) further confirmed that NRS-2002 is effective for predicting short-term outcomes (51), making it a valuable adjunct to MUST.

For gastric cancer, patients are prone to Sarcopenia, which directly affects surgical tolerance, postoperative recovery and prognosis. Therefore, tools that go beyond conventional nutritional screening and specifically assess muscle mass and function are of vital importance. Lu et al. (52) found that SARC-CalF provided strong diagnostic value (AUC 0.896) (52). Although self-screening tools such as MUST and MST are practical and reliable (53, 54), limitations exist in gastric cancer populations. To address this, Chen et al. (55) developed the SNRSGC tool for home-based self-assessment following gastrectomy (55).

In pancreatic cancer, Yu et al. (56) identified Controlling Nutritional Status (CONUT) as a strong prognostic indicator, while NRS-2002 was particularly predictive of mortality (HR 1.248; 95% CI, 1.155–1.348; *P* < 0.001) (56). The CONUT score reflects albumin depletion, lymphopenia, and hypocholesterolemia—hallmarks of pancreatic cancer malnutrition—whereas NRS-2002 integrates systemic disease burden. Menozzi et al. (57) recommended complementing screening with CT-based body composition analysis for surgical candidates (57, 58).

For head and neck squamous cell carcinoma (HNSCC), Tu et al. (59) demonstrated that PG-SGA is more sensitive preoperatively due to its detailed symptom tracking (e.g., dysphagia, anorexia), whereas NRS-2002 offers rapid, practical assessment postoperatively (59). Before the operation, the tumor itself caused dysphagia and insufficient intake. PG-SGA can record the subjective symptoms of patients in detail, with high sensitivity, and is suitable for formulating preoperative nutrition plans. After the operation, the risks shift to surgical trauma, stress response and fasting. The concise structure and assessment of “disease severity” of NRS-2002 make it more convenient for rapid implementation in the busy clinical environment after surgery. In esophageal and pharyngeal cancers, PG-SGA’s patient-reported components provide unique insights into swallowing difficulties, pain, and other functional impairments, enabling precision nutrition planning (60, 61).

### Critically ill and age-specific populations

In critically ill patients, the NUTRIC score demonstrated strong external validity for predicting mortality and nutritional intervention benefits (62). Further studies confirmed that mNUTRIC and NRS-2002 outperform other tools in predicting mortality and organ failure (63), although Rattanachaiwong et al. (64) highlighted their limited sensitivity for diagnosing severe malnutrition compared to ESPEN/ASPEN-based tools.

In pediatric oncology, Lovell et al. (65) identified significant resource constraints limiting nutrition care, while Gallo et al. (66) validated a novel pediatric screening tool with superior accuracy for detecting low muscle mass. These findings highlight an urgent need for age-specific and resource-adapted solutions.

In elderly cancer patients, a systematic evaluation identified six validated screening tools (MNA, MST, MUST, NRS-2002, NUTRISCORE, PG-SGA SF) with established cut-offs (67–69). The combination of MNA-SF (elderly-specific) and PG-SGA (oncology-specific) improved prediction of hospital stay length and readmission rates (69).

### Resource considerations and implementation barriers

Tool applicability is influenced by healthcare resources. In low-resource settings, simple, low-cost instruments like MUST and MST are practical due to minimal equipment requirements (53, 54). High-resource centers can leverage comprehensive multimodal assessments, integrating PG-SGA, CONUT, and CT imaging for precision care. However, implementation gaps remain a persistent challenge. Despite strong awareness of malnutrition’s impact, clinician adoption rates of validated tools remain low, and training gaps hinder consistency (70–72).

### Limitations of existing tools

Current instruments face three core limitations: (1) Subjectivity and recall bias: Tools like PG-SGA and MNA rely heavily on patient-reported intake and weight history, which may be inaccurate due to fatigue, cognitive decline, or treatment side effects (25). (2) Static, single-point evaluation: Tools such as NRS-2002 and MUST lack dynamic monitoring capacity, failing to capture rapid nutritional deterioration during therapy (73, 74). (3) Limited applicability in special populations: Obese cancer patients with sarcopenia often evade detection due to low-BMI thresholds (75, 76). Adjusted diagnostic cut-offs for weight and muscle loss in obesity have been proposed (77).

### Future research directions

Future research should focus on tool innovation, clinical integration, and international harmonization: (1) AI/ML-Driven Models: Kiss et al. (78) found that machine learning models based on GLIM criteria maintained predictive accuracy for clinical outcomes even when excluding muscle mass (78). Meanwhile, Wu et al. (79) developed an online machine learning tool to effectively predict malnutrition in colorectal cancer patients without weight loss data, identifying NRS-2002 as the most suitable screening method (79). Besides, tools like *MyCancerRisk* (80) and ML-based colorectal cancer prediction models (81) demonstrate how artificial intelligence can enhance precision screening, even in patients lacking classic weight-loss markers. (2) Personalized Protocols: Screening should be stratified by cancer type, baseline BMI, sarcopenia, and systemic inflammation to refine clinical accuracy (82). (3) Clinical Implementation: Strategies must emphasize clinician training, simplified workflows, and multidisciplinary data integration (82, 83). (4) Global Standardization: The absence of unified criteria remains a critical barrier (56, 84); internationally validated frameworks would enable consistent application and research comparability.

However, AI and ML integration faces significant challenges, including fragmented electronic health records, missing data, heterogeneous nutritional metrics, and risk of algorithmic bias. Transparency (Explainable AI), data protection, and human oversight remain essential (85). Future efforts should not only develop objective, dynamic tools but also optimize usability to improve adherence and facilitate cross-disciplinary collaboration, ultimately enabling nutrition assessment to become a seamless component of oncology care.

Cancer-related malnutrition screening and assessment tools exhibit significant heterogeneity in sensitivity, specificity, and clinical usability. Tool selection should be context-driven, considering cancer type, age, and resource availability (Table 6). Future directions emphasize AI-driven innovation, personalization, and international standardization to improve patient outcomes and integrate nutritional assessment into routine oncology practice.

**TABLE 6:** Tools for different cancer diseases and age groups.

Malnutrition screening and assessment tools for different cancer and age Groups				
Cancer type	Primary screening tool	Complementary/ alternative tools	Key considerations and rationale	
Pan-cancer/general inpatients	NRS-2002	MUST, MST	NRS-2002 is widely validated and effective for predicting short-term clinical outcomes. Its “disease severity” score is particularly relevant for cancer patients.	1. Static assessment: designed for a single-point assessment (e.g., admission), failing to capture dynamic deterioration during treatment (78, 79). 2. Subjectivity and bias: relies on patient self-report (weight change, intake), which can be inaccurate due to recall bias, fatigue, or cognitive impairment (72). 3. Lack of standardization: inconsistent application and intervention despite high awareness among staff (82, 84).
Colorectal cancer	MUST	NRS-2002	MUST shows a significant association with body composition and clinical outcomes. It effectively identifies risk from long-term consumption, side effects, and complications like obstruction.	–
Gastric cancer	SARC-CalF (for Sarcopenia)	MUST, MST, SNRSGC (for post-op home use)	Sarcopenia is highly prevalent. SARC-CalF demonstrated excellent clinical utility (AUC = 0.896). For post-operative self-screening at home, the specialized SNRSGC tool is recommended.	1. Tool limitations: tools like SARC-CalF require validation in broader populations. 2. Post-Op monitoring: lack of effective tools and protocols for dynamic monitoring after discharge.
Pancreatic cancer	CONUT and NRS-2002	PG-SGA, PNI	CONUT (based on albumin, lymphocytes, cholesterol) has the highest prognostic value for mortality. NRS-2002 complements it by accounting for disease-related factors (inflammation, metastasis). CT body composition analysis pre-surgery is highly recommended.	1. Confounding objective measures: albumin and lymphocytes used in CONUT are heavily influenced by the profound inflammatory response from the tumor itself, not purely nutritional status. 2. Complexity: the etiology of malnutrition in PC is multifactorial (malabsorption, inflammation, anorexia), making it difficult for any single tool to capture fully.
Head and neck cancer (HNSCC)	Pre-Op: PG-SGA Post-Op: NRS-2002	–	PG-SGA shows slightly higher sensitivity pre-operatively. NRS-2002 is more practical and suitable for use in the post-operative period.	1. PG-SGA subjectivity: patients with dysphagia, pain, and communication barriers may report symptoms and intake inaccurately, compromising PG-SGA accuracy (72). 2. Dynamic changes: nutrition status can deteriorate rapidly during RT/CT due to mucositis and taste changes, requiring more frequent monitoring than tool-based assessment allows.
Esophageal/pharyngeal cancer	CONUT, PNI, PG-SGA, NRS-2002	Serum albumin, body composition	Multiple tools are applicable. Serum albumin levels and the presence of sarcopenia (low muscle mass) are consistently important indicators of survival prognosis.	1. Impact of dysphagia: patient-reported food intake is highly unreliable, severely affecting the “intake change” items of PG-SGA and NRS-2002. 2. Need for dynamic monitoring: nutritional status changes rapidly from neoadjuvant therapy to surgery, but effective continuous monitoring protocols are lacking.
Critically ill cancer patients (ICU)	m-NUTRIC score and NRS-2002	–	The m-NUTRIC score is specifically designed for ICU settings and performs better in predicting mortality and organ failure. Note: It may have lower sensitivity for diagnosing severe malnutrition alone.	1. Diagnostic sensitivity: the NUTRIC score has low sensitivity for diagnosing severe malnutrition (73). 2. Operational complexity: indicators required for m-NUTRIC (e.g., IL-6) are not routinely measured in all ICUs, limiting its broad application.
**Malnutritional Screening and Assessment Tools for different age groups**				
**Population group**	**Recommended screening tools**	**Recommended assessment/diagnostic tools**	**Key points and explanations**	**Challenges and limitations**
Adult oncology outpatients	● MNA (≤ 23.5) ● MST (≥ 2, including patient-led) ● MUST (≥ 1 or ≥ 2) ● NRS-2002 (≥ 2 or ≥ 3) ● NUTRISCORE (≥ 5) ● PG-SGA SF (≥ 7 or ≥ 8)	A more comprehensive assessment by a dietician is typically required for formal diagnosis.	A systematic review identified these six tools as effective at specific cut-off points. Tool selection should be based on population characteristics (e.g., cancer type, stage, treatment modality).	1. Subjectivity and recall bias: tools like PG-SGA and MNA rely on patient self-report (e.g., dietary intake, weight change), which can be inaccurate due to fatigue, cognitive impairment, or treatment side effects. 2. Static assessment: tools like NRS-2002 and MUST are designed for a single-point assessment (e.g., admission) and fail to capture the dynamic deterioration of nutritional status during treatment. 3. Lack of standardization: despite high awareness, a lack of standardized protocols for screening and management leads to inconsistent practice in clinical settings.
Pediatric cancer patients	● Custom screening tool (66) ● Other validated tools (not explicitly named)	–	Resource limitations and inadequate screening hinder nutritional intervention. The custom tool by Gallo et al. demonstrated superior accuracy in identifying low muscle mass, providing a valuable reference for pediatric clinical practice.	Lack of resources and standardization: this area is hindered by limited resources and inadequate screening/assessment, which blocks effective nutritional intervention.
Geriatric cancer patients	Recommended tools: ● MNA or MNA-SF (ESPEN recommendation) ● PG-SGA SF ● Combined tool: MNA-SF + PG-SGA	Diagnostic tools: ● SGA ● PG-SGA ● GLIM criteria	● Lower MNA-SF scores are significantly associated with longer hospital stays and higher mortality. ● Combined use of MNA-SF (for geriatrics) and PG-SGA (for oncology) may better predict length of hospital stay and readmission risk in elderly patients with solid tumors.	Shares common limitations:also faces challenges of subjectivity, static assessment, and lack of standardization in clinical application.
Obese cancer patients	–	Diagnostic tools: ● GLIM criteria (currently with limitations)	–	1. Poor applicability: current phenotypic criteria (e.g., low BMI) do not apply to obese patients, who may have sarcopenia (sarcopenic obesity). 2. Inaccurate criteria: this leads to inaccuracies with tools like GLIM in this population. Solution: thresholds for appropriate weight loss and muscle mass loss specific to obesity need to be defined to update the criteria.

A quasi-systematic review was performed; therefore, results do not represent an exhaustive search of the literature. NRS-2002, Nutritional Risk Screening 2002; MUST, Malnutrition Universal Screening Tool; MST, Malnutrition Screening Tool; SARC-CalF, strength, assistance in walking, rise from a chair, climb stairs, falls combined with calf circumference; SNRSGC, self-screening tool for nutrition risk in patients with gastric cancer after gastrectomy; CONUT, Controlling Nutritional Status; PG-SGA, Patient-Generated Subjective Global Assessment; PNI, Prognostic Nutritional Index; m-NUTRIC score, modified nutrition risk in the critically ill score; MNA, Mini Nutritional Assessment; NUTRISCORE, Nutrition Risk Score; PG-SGA SF, Patient-Generated Subjective Global Assessment Short Form; MNA-SF, Mini-Nutritional Assessment Short-Form; SGA, subjective global assessment; GLIM, The Global Leadership Initiative on Malnutrition. This table provides a comprehensive overview of recommended nutritional screening and assessment tools tailored to specific cancer types and different age populations. The top section details tool selection based on cancer type (e.g., MUST for colorectal, CONUT and NRS-2002 for pancreatic, SARC-CalF for gastric), highlighting the primary rationale for each choice and key limitations such as subjectivity, static nature of tools, and need for dynamic monitoring. The bottom section addresses tool application across age groups (adults, pediatrics, geriatrics, and obese patients), outlining validated tools, key considerations, and population-specific challenges.

## Conclusion

Malnutrition remains a prevalent and clinically significant complication in oncology, with profound implications for treatment tolerance, recovery, and overall survival. In recent years, the development and refinement of cancer-specific nutritional screening and assessment tools have become a focal point in clinical nutrition and oncologic care. As evidence continues to underscore the detrimental effects of cancer-related malnutrition, research efforts have increasingly shifted toward early detection strategies, integrating simplified yet comprehensive tools to optimize patient outcomes. Emerging trends emphasize the combination of multiple validated instruments with novel technologies—including artificial intelligence, machine learning, and digital health platforms—to improve diagnostic precision, enhance clinical applicability, and facilitate timely, personalized nutritional interventions.
